# PrePex Male Circumcision: Follow-Up and Outcomes during the First Two Years of Implementation at the Rwanda Military Hospital

**DOI:** 10.1371/journal.pone.0138287

**Published:** 2015-09-23

**Authors:** Albert Ndagijimana, Pacifique Mugenzi, Dana R. Thomson, Bethany Hedt-Gauthier, Jeanine U. Condo, Eugene Ngoga

**Affiliations:** 1 University of Rwanda College of Medicine and Health Sciences School of Public Health, Kigali, Rwanda; 2 Rwanda Military Hospital, Kigali, Rwanda; 3 Department of Global Health and Social Medicine, Harvard Medical School, Boston, Massachusetts, United States of America; University of Pittsburgh, UNITED STATES

## Abstract

**Background:**

PrePex Male Circumcision (MC) has been demonstrated as an effective and scalable strategy to prevent HIV infection in low- and middle-income countries. This study describes the follow-up and outcomes of clients who underwent PrePex MC between January 2011 and December 2012 with weekly follow-up at the Rwanda Military Hospital, the first national hospital in Rwanda to adopt PrePex.

**Methods:**

Data on570 clients age 21 to 54 were extracted from patient records. We compared socio-demographic and clinical characteristics, the operator's qualification, HIV status, pain before and after device removal, urological status, device size and follow-up time between clients who were formally discharged and those who defaulted. We reported bivariate associations between each covariate and discharge status, number of people with adverse events by discharge status, and time to formal discharge or defaulting using life table methods. Data were entered into Epidata and analyzed with Stata v13.

**Results:**

Among study participants, 96.5% were circumcised by non-physician operators, 85.4%were under 30years, 98.9% were HIV-negative and 97.9% were without any urological problems that could delay the healing time. Most (70.7%) defaulted before formal discharge. Pain before (p<0.001) and after PrePex device removal (p = 0.001) were associated with discharge status, although very few cases were reported, and pain was more commonly missing among defaulters. Twenty-seven adverse events were reported (7 formally discharged, 20 defaulters). Median follow-up time was seven weeks among formally discharged and six weeks among defaulters (p<0.001).

**Conclusion:**

Given that all socio-demographic and most clinical characteristics were not associated with defaulting, we hypothesize that clients stopped returning once they determined they were healed. We recommend less frequent follow-up protocols to encourage clinical visits until formal discharge. Based on these results and recommendations, we believe PrePex MC is a practical circumcision strategy in Rwanda and in sub-Saharan Africa.

## Introduction

Surgical male circumcision(MC) is among the oldest surgical procedures worldwide[1], motivated by various cultural, religious, hygienic and medical reasons [2]. Since the 1960s, MC has been one of the most effective strategies to prevent sexually transmitted infections and to address complications from sexual intercourse[1,3]. More recent observational and randomized controlled trials have shown that MC reduces the risk of HIV infection among adult males by at least 50% [4,5]. The vast majority of all new HIV infections occur in sub-Saharan Africa and 70% are due to heterosexual intercourse[4]. In this region, poverty, among other factors, limits access to HIV prevention and treatment. As a result, WHO/UNAIDS recommends universal MC as a population-level HIV prevention strategy in countries with high HIV prevalence driven by heterosexual transmission and low male circumcision prevalence[4,6].

To have an impact on HIV transmission, WHO/UNAIDS set a target of 20 million men for male circumcision in Africa by 2015[7]. Although surgery has been the most commonly used MC procedure in adults, risks include bleeding, infections, mortality from anesthesia, and in rare cases, complications that may lead to penile damage or sexual impotence[1,4]. Surgical MC is a relatively time-intensive procedure performed in a sterile environment by a physician using many instruments, with intensive care during the wound period[3,4].For these reasons, surgical MC is not ideal for mass intervention in adult men in Africa due to limited resources and few equipped health facilities and physicians[8].

In Rwanda, 3% of adults are infected with HIV, and only 13% of Rwandan men were circumcised in 2010[9]. In 2009, the Rwanda Ministry of Health set the target of 2 million adult male circumcisions by the end of 2012[10].With only 1 physician per 16,000 inhabitants[11], the Rwandan government considered alternatives to surgical MC including the Shang Ring device [12] and the circular cutter with stapled anastomosis [13].The Ministry of Health opted for PrePex, a quick, non-surgical procedure that does not require highly qualified staff or a sterile environment [14]. With three clinical trials in Rwanda, the procedure was pre-qualified in March 2012 by the WHO[10].Rwanda was the first country to adopt PrePex in the public health setting on a mass scale.

There are several benefits to PrePex including the ability to shift the MC procedures to less-trained staff, which makes the procedure cost-effective and scalable[15], even in high resource settings[16].Further, the minimal invasiveness and risk make the procedure acceptable to beneficiaries[17]. However, there is little research describing the implementation of PrePex in a real world setting, outside of a research study environment. Rwanda Military Hospital (RMH), a referral hospital in the country’s capital city, Kigali, which serves military members and civilians (80% of patients) was the first hospital in the country to offer PrePex MC procedure starting in 2011. The goal of this paper is to describe the follow-up and outcomes of the first clients who underwent PrePex MC procedure at Rwanda Military Hospital (RMH).

## Methods

### Study design and population

This descriptive cross-sectional study included a retrospective review of records collected on men age 21 to 54 receiving PrePex MC between January 2011 and December 2012 at RMH. To receive PrePex MC, the client needed to demonstrate an ability to understand the study procedures and requirements, comprehend and freely give informed consent, agree to abstain from sexual intercourse following medical orders and agree to return to the health care facility for follow-up visits.

### PrePex procedure

The PrePex MC procedure is carried out in a clean environment, usually by a nurse, and takes approximately five minutes. The device is comprised of three rings: the placement ring, inner ring, and elastic ring. The procedure is comprised of the following steps: the client’s penis is sized to select the appropriate device size, the operator disinfects and marks the foreskin where the device should be placed, the elastic band is loaded onto the placement ring, the placement ring is slid over the penis, the inner ring is placed inside of the foreskin and adjusted to the circumcision marking line, and finally the elastic ring is rolled from the placement ring into the inner ring pressing the foreskin tightly. The procedure does not involve cutting live tissue, anesthesia or sutures. After the PrePex ring is placed, the client continues to wear his clothes as usual and embarks on his normal life. The ring is removed seven days after its placement when the foreskin is necrotic. The dead tissue is removed with scissors, and any local wound from the device removal is treated. In this study, the client was requested to return for weekly follow-up visits until the wound was judged completely healed based on physical examination by a trained operator; when the client was determined to have only a scar remaining he was formally discharged from the program. The client was also instructed to return for any urgent issues, regardless of the weekly follow-up visit schedule.

### Data collection and analysis

This study used clinical records at RMH. Records were entered into Epidata by trained data entry clerks under supervision of the University of Rwanda-College of Medicine and Health Sciences-School of Public Health (UR-CMHS-SPH). Data quality was checked by looking for outliers and comparing electronic and paper forms in outlier records.

We first explored the following factors in clients who were formally discharged versus those who defaulted before formal discharge: age in years, qualification of the operator (nurse or physician), HIV status, urology history and status, level of pain reported before and after device removal, adverse events any time after device placement, and the PrePex device size. Relationships were assessed using the Fisher’s Exact test, and the follow-up time among the two groups was measured using two-sample Wilcoxon-Mann-Whitney test; both at the α = 0.05 significance level. Frequencies of adverse events (for all patients) and weeks of healing time (for patients that formally discharged, the time between device placement and formal discharge) were reported. The cumulative probability of the PrePex wound being healed or time to defaulting was calculated using a life table approach. All analyses were completed in Stata v13.

### Ethics statement

At the first encounter, an operator explained the clinical procedure to the client and answered any questions. Then the operator presented a written consent form in Kinyarwanda to the client, and verbally explained its content: that information collected during medical visits related to the PrePex procedure would be used in research, no identifying information about the client would be published, and that the client had the option to receive the surgical procedure available countrywide in case he did not agree to participate in this study. A client provided a written signature and checked a box for whether information could be shared, and he was provided a copy of this signed consent form. The operator marked a yes/no check box in the medical record indicating that consent was obtained, and a copy of the signed consent form was provided to the client. Clients’ confidentiality was secured by keeping paper records in a locked cupboard with access only by authorized nurses. Data were entered in an electronic database by trained data entry clerks at UR-CMHS-SPH, and no personal identifiers were electronically recorded. All electronic data were stored on password protected computers held by the study research team. This study protocol was approved by UR-CMHS-SPH Institutional Review Board (ethical clearance number: 028/UR/CMHS/SPH/2014).

## Results

Between January 2011 and December 2012, 639 men were screened for MC, 66 men did not meet inclusion criteria, and 570 men received MC with PrePex at RMH. Among these clients, 167 (29.3%) were formally discharged and 403 (70.7%) defaulted before formal discharge. Most clients were between 21 and 24 years old (57.7%) or 25 and 29 years (27.7%), and nearly all clients were circumcised by a non-physician operator (97.0% and 96.3%, respectively, among formally discharged and defaulters). Most men were HIV negative (99.4% among formally discharged and 98.8% among defaulters) and had no evidence of urological problems that could delay their healing and formal discharge (97.0% among formally discharged and 98.3% among defaulters). Very few men reported pain either before the device removal (0.5%) or after the device removal (0.7%). Among all clients, 37.5% were fitted with the device size B (28 mm), although, there was no statistical significant between the device size and defaulting status (p = 0.725). Pain before (1.2% versus 0.3%) and after the device removal (0.6% versus 0.7%) were similar, but more clients were missing pain data among defaulters versus clients who were formally discharged (p<0.001). The median follow-up time from device application date was seven weeks (IQR: 5, 9) and six weeks (IQR: 1.8) respectively among formally discharged and defaulter clients (p = 0.001) (Table 1).

**Table 1:** Bivariate associations between discharge status and socio-demographic and clinical characteristics at Rwanda Military Hospital, 2011 (N = 570).

Variables		Formally discharged		Defaulted before formal discharge		p-value	Total	
		n	%	n	%		N	%
Overall		167	29.3	403	70.7		570	100
Age						0.555		
	21–24	97	58.1	232	57.6		329	57.7
	25–29	50	29.9	108	26.8		158	27.7
	30–34	11	6.6	44	10.9		55	9.7
	35–39	6	3.6	14	3.5		20	3.5
	40–44	3	1.80	3	0.7		6	1.1
	45–54	0	0.0	1	0.3		1	0.2
	Missing	0	0.0	1	0.3		1	0.2
	Total	167	100.0	403	100.0		570	100.0
Operator's qualification						0.225		
	Physicians	4	2.4	5	1.2		9	1.6
	Non physicians	162	97.0	388	96.3		550	96.5
	Missing	1	0.6	10	2.5		11	1.9
	Total	167	100.0	403	100.0		570	100.0
HIV status						0.435		
	Positive	1	0.6	5	1.2		6	1.1
	Negative	166	99.4	398	98.8		564	98.9
	Total	167	100.0	403	100.0		570	100.0
Urology history						0.256		
	Urology problem	5	3.0	7	1.7		12	2.1
	No urology problem	162	97.0	396	98.3		558	97.9
	Total	167	100.0	403	100.0		570	100.0
Pain Before Device Removal						<0.001		
	Yes	2	1.2	1	0.3		3	0.5
	No	148	88.6	202	50.1		350	61.4
	Missing	17	10.2	200	49.6		217	38.1
	Total	167	100.0	403	100.0		570	100.0
Pain After Device Removal						0.001		
	Yes	1	0.6	3	0.7		4	0.7
	No	166	99.4	378	93.8		544	95.4
	Missing	0	0.0	22	5.5		22	3.9
	Total	167	100.0	403	100.0		570	100.0
Adverse Event						0.189		
	Yes	7	4.2	20	4.9		27	4.7
	No	160	95.8	375	93.1		535	1.4
	Missing	0	0.0	8	2.0		8	93.9
	Total	167	100.0	403	100.0		570	100.0
Device size						0.725		
	A(26 mm)	45	26.9	99	24.6		144	25.3
	B(28 mm)	61	36.5	153	38.0		214	37.5
	C(30 mm)	42	25.2	109	27.1		151	26.5
	D(32 mm)	15	9.0	30	7.4		45	7.9
	E(34 mm)	1	0.8	8	2.0		9	1.6
	Missing	3	1.8	4	1.0		7	1.2
	Total	167	100.0	403	100.0		570	100.0
Follow-up time(median, IQR)		7	5,9	6	1,8	<0.001		

Few adverse events were recorded (four cases of diffuse edema, four of bleeding, five of wound infection, three of productive exudate and eleven others), of which20 (4.9%) were among defaulters and seven (4.2%) were among formally discharged (Table 2). Most clients healed between six and eight weeks (68.3%), very few (6.6%) required more than eight weeks to heal; and seven adverse events occurred after the sixth week (Table 3).Most clients defaulted between six and eight weeks (42.2%), few defaulted between nine and ten weeks (0.7%); and four adverse events occurred between the second and fourth weeks, and 16 after the sixth week (Table 4).

**Table 2:** Number of adverse events among those who defaulted and remained.

Types of adverse events	Formally discharged	Defaulted before formal discharge
Diffuse edema	1	3
Bleeding	1	3
Wound infection	1	4
Productive exudate	1	2
Other	3	8
Total	7(4.2% of 167 clients)	20(4.9% of 403 clients)

Note–one client may have multiple adverse events.

**Table 3:** Time and cumulative probability to formal discharge/healing(N = 167).

Week interval when the wound was certified healed	Total by the interval time	Number with wound healing	Cumulative probability	95% CI		Timing and number of adverse events
3–4 weeks	167	1	0.0600	0.0008	0.0417	
4–5 weeks	166	1	0.0120	0.0030	0.0470	
5–6 weeks	165	13	0.0898	0.0551	0.1446	
6–7 weeks	152	57	0.4311	0.3601	0.5098	4
7–8 weeks	95	57	0.7725	0.7065	0.8327	2
8–9 weeks	38	27	0.9341	0.8897	0.9651	
9–10 weeks	11	9	0.9880	0.9609	0.9976	
10–11 weeks	2	2	1.0000	-	-	1

**Table 4:** Time and cumulative probability to defaulting (N = 403).

Week interval when the client defaulted	Total by the interval time	Number of defaulters	Cumulative probability	95% CI		Timing and number of adverse events
1–2 weeks	396	30	0.0758	0.0536	0.1066	
2–3 weeks	366	58	0.2222	0.1844	0.2665	2
3–4 weeks	308	31	0.3005	0.2579	0.3483	2
4–5 weeks	277	25	0.3636	0.3184	0.4131	
5–6 weeks	252	44	0.4747	0.4269	0.5251	
6–7 weeks	208	88	0.6970	0.6513	0.7415	7
7–8 weeks	120	82	0.9040	0.8726	0.9305	6
8–9 weeks	38	35	0.9924	0.9793	0.9979	3
9–10 weeks	3	3	1.0000	-	-	

## Discussion

The results of this real-world scale-up of PrePex MC at RMH are promising for the scale-up of PrePex in other similar settings. In the first two years, 570 adult men were circumcised with PrePex, primarily by non-physician staff. The few instances of adverse events and the recorded healing times were consistent with larger clinical studies on PrePex where the median time for healing was five to six weeks [18]. PrePex takes on average one to two weeks longer to heal than surgical MC[19,20] or Shang ring[21].

The biggest challenge in this setting was the high rate of defaulting; a majority of patients discontinued weekly follow-up visits before being formally discharged from the program. The high rate of default during and after the sixth week might be because clients perceived that they were healing properly and they did not feel a need to return for clinical assessments. We suspect that a client with a significant problem would have come back for clinical follow-up. Given the high rate of default before formal discharge, RMH is now using a protocol with less frequent follow-up visits to improve adherence without compromising clinical outcomes.

The only socio-demographic or clinical difference between the groups was the amount of missing data in reported pain before and after device removal. One explanation for this difference in missing data is that clients who defaulted tended to be the same types of clients who do not respond to all questions during removal of the PrePex device. Another explanation is that clients who were not asked about pain during device removal felt less rapport with clinical staff and were more likely to default, though ongoing supervision of clinical staff throughout the study makes this explanation unlikely.

The findings about adverse events among defaulters suggest that there were not substantial differences in number, type, and timing of adverse events and experiences of pain between the two groups which might drive someone to default. Other studies assessing the efficacy of PrePex procedure in a more formal study environment in Uganda and Rwanda also found few adverse events among clients[14,17,18,22] and it is reassuring to see this continue in a real-world setting. Given the low rate of adverse events and large amount of defaulting, implementers should consider fewer follow-up visits.

There are some limitations to this study. HIV, urological problems and adverse events were all rare, and our sample may have been too small to detect differences between the defaulter and non-defaulter groups. However, there is no indication of a difference between the groups and we hypothesize that individuals are defaulting before formal discharge because they are healing well. This should be studied more in the future. Additionally, although we collected information about type of adverse event, information about the severity of adverse events was not collected and should be collected in future studies. Finally, RMH is a national referral hospital with different services and infrastructure compared to district hospitals and health centers where this procedure is mostly likely to be scaled. However, as PrePex procedure is performed in a clean environment, with non-physicians and without anesthesia, we expect that outcomes and few complications observed in this study would be replicated in other clinical settings in Rwanda and in sub-Saharan Africa.

Rwanda has been successful in implementing PrePex as a non-surgical MC procedure, a strategy to supplement surgical MC to achieve the Ministry of Health target of two million adult male circumcisions by 2012.However, the number of men circumcised in this two years period at RMH is small compared to the two million targeted men; as of 2014, PrePex is only implemented at RMH health facilities in Rwanda and during army weeks and RMH outreach activities. Given these promising results, and that the procedure can be performed in a non-sterile environment with a non-physician with very few adverse events, we strongly encourage scale up of PrePex MC at all district hospitals in Rwanda. However, more evaluation of post PrePex follow-up protocols should be conducted to encourage clinical monitoring of MC clients until full documented healing.
